# The outer membrane phospholipase A is essential for membrane integrity and type III secretion in *Shigella flexneri*

**DOI:** 10.1098/rsob.160073

**Published:** 2016-09-21

**Authors:** Xia Wang, Feng Jiang, Jianhua Zheng, Lihong Chen, Jie Dong, Lilian Sun, Yafang Zhu, Bo Liu, Jian Yang, Guowei Yang, Qi Jin

**Affiliations:** MOH Key Laboratory of Systems Biology of Pathogens, Institute of Pathogen Biology, Chinese Academy of Medical Sciences and Peking Union Medical College, People's Republic of China

**Keywords:** *Shigella flexneri*, phospholipase A, cell membranes, host cell invasion

## Abstract

Outer membrane phospholipase A (OMPLA) is an enzyme located in the outer membrane of Gram-negative bacteria. OMPLA exhibits broad substrate specificity, and some of its substrates are located in the cellular envelope. Generally, the enzymatic activity can only be induced by perturbation of the cell envelope integrity through diverse methods. Although OMPLA has been thoroughly studied as a membrane protein in *Escherichia coli* and is constitutively expressed in many other bacterial pathogens, little is known regarding the functions of OMPLA during the process of bacterial infection. In this study, the proteomic and transcriptomic data indicated that OMPLA in *Shigella flexneri*, termed PldA, both stabilizes the bacterial membrane and is involved in bacterial infection under ordinary culture conditions. A series of physiological assays substantiated the disorganization of the bacterial outer membrane and the periplasmic space in the *ΔpldA* mutant strain. Furthermore, the *ΔpldA* mutant strain showed decreased levels of type III secretion system expression, contributing to the reduced internalization efficiency in host cells. The results of this study support that PldA, which is widespread across Gram-negative bacteria, is an important factor for the bacterial life cycle, particularly in human pathogens.

## 1. Introduction

The outer membrane proteins (OMPs) of Gram-negative bacteria are unique membrane proteins that generally contain a β-barrel fold and range in size from 8 to 26 strands [1,2]. OMPs are synthesized in the cytoplasm, and transported to and inserted into the outer membrane (OM), an asymmetrical bilayer comprising lipopolysaccharide (LPS) in the outer leaflet and different types of phospholipids (PLs) in the inner leaflet [3,4]. Several OMPs provide a variety of functions, such as signal transduction, catalysis, immunity and pathogenicity [5–8].

Outer membrane phospholipase A (OMPLA) comprises a family of β-barrel proteins embedded in the OM of bacteria that hydrolyse membrane PLs and remove the ester bonds at the stereochemical numbering (sn) positions sn-1 (first carbon) or sn-2 (second carbon) from the glycerophosphodiester backbone of both PLs and lysophospholipids [9,10]. The crystal structure of the OMPLA isolated from *E. coli* indicated that this enzyme is a serine hydrolase with a His142-Ser144-Asn156 catalytic triad located on the exterior of the β-barrel. The activity of this enzyme is regulated by reversible dimerization and requires calcium as a cofactor [11,12].

OMPLA, in combination with other bacterial components, plays a pivotal role in the maintenance of the OM structural integrity and stability [13]. In *E. coli*, OMPLA is constitutively expressed, and computational studies indicate that PldA interacts with LPS and maintains lipid asymmetry in outer membranes under homoeostatic conditions [13,14]. Nevertheless, there is scarce experimental evidence addressing whether and how the constitutively expressed PldA affects the OM integrity under normal growth conditions. In some human pathogens, OMPLA also functions as a virulence determinant with various mechanisms. For example, in *Helicobacter pylori* OMPLA determines the initial fitness for colonization and subsequent niche adaptation, and in *Campylobacter coli* this enzyme is considered as a major haemolytic factor [15–17].

OMPLA is also constitutively expressed in *Shigella flexneri* (termed PldA), which is the leading cause of bacillary dysentery in humans [18]. Genome sequencing revealed that *S. flexneri* possesses a mega virulence plasmid, which encodes the invasion plasmid antigen IpaACDB and the Mxi-Spa-type III secretion system (T3SS) [19,20]. *Shigella flexneri* uses the T3SS, a needle-like structure, to invade epithelial cells from the basolateral side and inject effectors into the host cell cytoplasm. These effectors can influence host cellular function, subvert host–cell signalling pathways and regulate inflammatory responses [21–24]. Upon invasion, *S. flexneri* lyses the vacuole membrane, replicates within the host cell cytoplasm and spreads to adjacent cells [25,26]. In addition to the plasmid-borne virulence determinants, many metabolic pathways and OMPs are important for pathogen invasion, intracellular growth and cell-to-cell spread, including carbon metabolism pathways, the synthesis of amino acids and nucleotides, OmpA and OmpC, and other molecules [5,6,27,28]. Previously, proteomic analyses revealed that intracellular *S. flexneri* exhibits increased expression levels of PldA protein [29], indicating that PldA is probably involved in the pathogenicity towards host cells. However, there has been no experimental evidence showing that PldA regulates *S. flexneri* invasion and pathogenesis.

An understanding of shigellosis pathogenesis is important for vaccine development and treatment. In this study, we report that *Shigella* PldA plays an important role not only in OM structural integrity and stability but also in *S. flexneri* internalization into epithelial host cells. Through comprehensive proteomic and transcriptomic comparisons between wild-type (WT) and *pldA* knockout *S. flexneri* strains, we demonstrated that PldA deficiency affected the integrity of the bacterial membrane, leading to the generation of less vigorous pathogens. Furthermore, the PldA deletion also affected the T3SS-related invasion efficiency, resulting in the decreased pathogenicity of *S. flexneri*. Moreover, intracellular *S. flexneri* delivers PldA into the host cell cytosol. Furthermore, homologues of PldA are encoded in diverse bacterial pathogens, including *H. pylori* and *C. coli*, suggesting that these proteins are probably important in host pathogenicity. Taken together, these findings determine multiple mechanisms for the contribution of PldA to the membrane integrity and pathogenicity of *S. flexneri*.

## 2. Results

### 2.1. *Shigella flexneri* wild-type and *ΔpldA* mutant strains show distinct secretomic components

**Figure 1.**
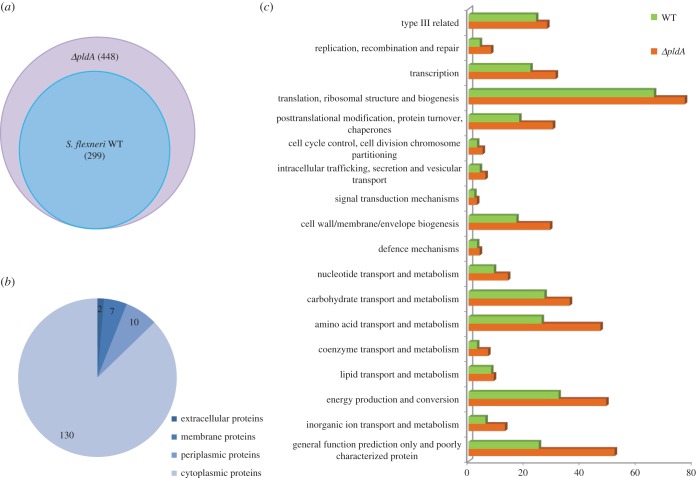
Identification of proteins in the culture supernatants of *S. flexneri* WT and *ΔpldA* strains using MS. (*a*) Venn diagram representing the relative abundance of proteins detected in the culture supernatant of the *ΔpldA* strain compared with the WT strain. In total, 299 proteins were identified in both WT and *ΔpldA* supernatant proteomes, while 149 proteins were unique to the *ΔpldA* supernatant proteome. (*b*) Pie chart predicting the cellular distribution of proteins unique to the *ΔpldA* supernatant proteome. Protein localization was predicted using the bioinformatics algorithms PSORTb, SignalP, TMHMM and KEGG. (*c*) Comparative proteome of WT and *ΔpldA* culture supernatants. Proteome mining and classification into functional categories was based on the database of Clusters of Orthologous Groups (http://www.ncbi.nlm.nih.gov/COG/) and the biological processes in which they are involved.

Previous studies have shown that PldA is located in the bacterial OM and interacts with the OM components [11–13]. To investigate the role of PldA protein on the cellular membrane, we applied proteomic assays to examine the secretomes of the WT and *ΔpldA* mutant strains. Because WT and *ΔpldA* mutant strains exhibited similar growth rates (electronic supplementary material, figure S1), we analysed the components precipitated from the culture supernatant of WT and mutant strains in the exponential growth phase using mass spectrometry (MS). The majority of the secreted proteins in samples from each of the three replicates were present in the supernatants from each of the strains and showed good technical and biological reproducibility. By setting a cut-off of two or more unique peptides per protein, we identified 448 secreted proteins in the *ΔpldA* mutant supernatant and 299 secreted proteins in the WT supernatant (figure 1*a* and electronic supplementary material, table S2). Among the 149 additional proteins secreted by the mutant, the majority were cytoplasmic proteins (130 proteins). The remaining proteins included 10 periplasmic proteins, 7 membrane proteins and 2 proteins predicted to have extracellular locations (figure 1*b*). Additionally, the unique proteins in the *ΔpldA* supernatant corresponded to functional categories associated with ‘posttranslational modification/protein turnover/chaperones’, ‘cell wall/membrane/envelope biogenesis’, ‘macromolecule transport and metabolism’ and ‘poorly characterized proteins’ (figure 1*c*). Thus, the profound changes in the *S. flexneri ΔpldA* mutant secretome implied that abolishing the *S. flexneri pldA* gene could lead to the impairment of cell wall integrity, decreased membrane stability and the subsequent leakage of the bacterial cytoplasmic components, which might affect the invasion capability of this bacterial pathogen.

### 2.2. PldA is required for maintaining proper bacterial morphology

**Figure 2.**
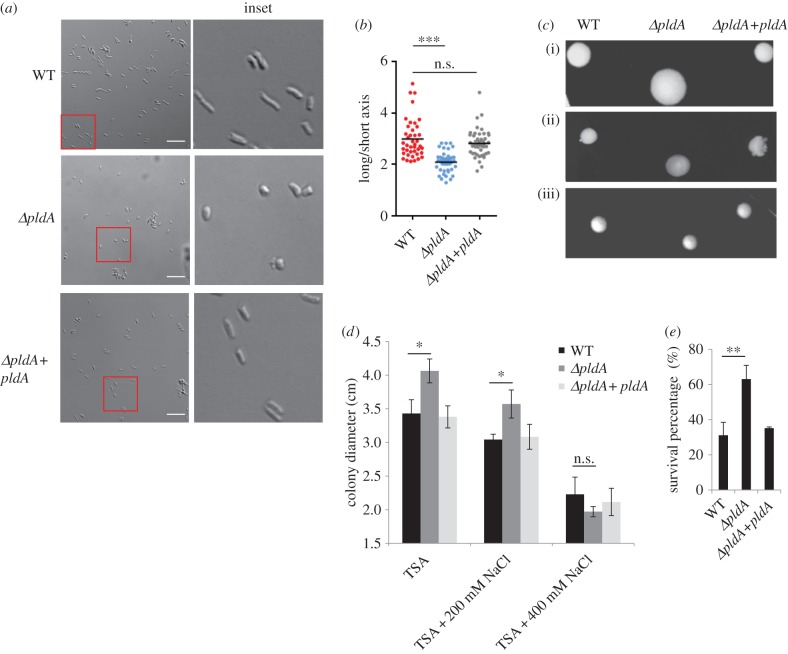
*pldA* deficiency alters *S. flexneri* cell morphology. (*a*) Morphology of the indicated *S. flexneri* strains was measured using confocal microscopy at 40× or 100× magnification after growth in TSB medium to exponential phase. Scale bars, 10 µm. (*b*) Relative ratio between the long and short axis of bacteria in (*a*) were calculated. The data were analysed using confocal microscopy software. More than 40 bacteria in each group were assessed for statistical analysis. (*c*) The colony morphology of indicated *S. flexneri* strains grown on TSA plates containing different salt concentrations: (i) TSA, (ii) TSA + 200 mM NaCl and (iii) TSA + 400 mM NaCl. (*d*) Average colony diameters of indicated strains as shown in (*c*) were measured. The error bars represent ±s.e.m. of 30 colonies (*n* = 3). (*e*) SDS-EDTA permeability assays. Indicated bacterial strains grown in the exponential phase were treated with 0.5% SDS in the presence of 1.5 mM EDTA; the OD600 for each strain was monitored, and lysis was normalized to a buffer control. The error bars represent ±s.e.m. (*n* = 3). *p < 0.05; **p < 0.01; ***p < 0.001; n.s., not significant.

The secretome profile of the WT and the *ΔpldA* mutant indicated that PldA played a critical role in maintaining the cell wall architecture. To further investigate the function of PldA in maintaining the *S. flexneri* OM, we examined the bacterial shape of *S. flexneri* WT and *ΔpldA* mutant using confocal microscopy. Compared with the rod-shaped WT strain, the *ΔpldA* mutant exhibited a more spherical, round shape (figure 2*a*,*b*). The complementation of PldA in the *ΔpldA* mutant restored the rod-like shape (figure 2*a*,*b*). This distinct shape of the *ΔpldA* mutant presumably reflected the disorganized structure of the bacterial OM. The spherical appearance in the mutant therefore suggests that PldA is required for the maintenance of the rod-like shape of *S. flexneri*, typically defined by the mechanically stiff exoskeletal cell wall [30].

Bacterial cell envelope stability is also associated with the morphology of the bacterial colonies and the osmotic stress response. To further substantiate these findings, we assayed the bacterial colony morphology under different osmotic pressures. Under normal culture conditions, the deletion of *pldA* gene resulted in the formation of larger colonies than those observed with the WT strain (figure 2*c*,*d*). Interestingly, the presence of 400 mM salts in the medium reduced the colony diameter of the *ΔpldA* mutant to a size similar to that observed in the WT colony (figure 2*c*,*d*), indicating that salt concentration resulted in more balanced osmotic pressures on both sides of the cell membrane in the mutant. These data suggested that the *ΔpldA* mutant was more fragile and sensitive to osmotic pressures, probably reflecting the loss of an intact OM. The complemented *ΔpldA* strain exhibited a similar colony size as the WT strain (figure 2*d*). Moreover, in SDS-EDTA permeability assays, the *ΔpldA* mutant was more resistant to SDS lysis in the presence of EDTA compared with the WT strain. The complemented *ΔpldA* strain displayed similar phenotype as the WT strain (figure 2*e*). The OM profile was also analysed by MS identification, and approximately 30% of OMPs were undetectable in the *ΔpldA* strain (electronic supplementary material, table S3). These results suggested a disorganized OM structure in the mutant bacterium. Taken together, these results support the conclusion that PldA is required for maintaining bacterial morphology through the maintenance of the integrity of the bacterial OM.

### 2.3. Loss of PldA results in antibiotic and acid resistance

**Figure 3.**
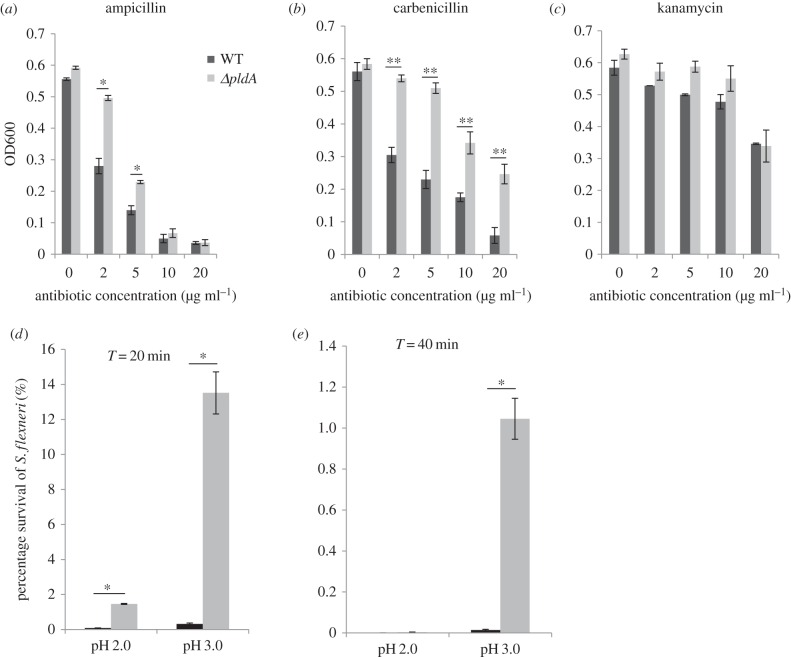
*pldA* affects β-lactam antibiotic and acid resistance*.* (*a*–*c*) The OD600 of indicated *S. flexneri* strains after 24 h of growth in TSB medium in the presence of β-lactam or non-β-lactam antibiotics (ampicillin, carbenicillin, kanamycin). All antibiotic concentrations are below the MICs for the bacteria. (*d*,*e*) Survival rates of *S. flexneri* (exponential phase grown cultures) under acid shock at pH 2.0 or pH 3.0 for 20 and 40 min. The error bars represent ±s.e.m. (*n* = 3), *p < 0.05; **p < 0.01.

Because the *pldA* deletion disrupted the integrity of the bacterial OM, we hypothesized that this disruption would further impair the organization of the periplasmic space. To examine this hypothesis, we determined the susceptibility of the WT and *ΔpldA* mutants to antibiotics acting through different antimicrobial mechanisms. When treated with antibiotics targeting bacterial cell walls, such as ampicillin or carbenicillin, the *ΔpldA* mutant exhibited approximately 50% higher survival rate than the WT strain (figure 3*a*,*b*). This effect might reflect the loss of natural targets in the periplasmic space. However, treatments with kanamycin, gentamicin or streptomycin, which interrupt intracellular protein synthesis, resulted in the similar survival of WT and *ΔpldA* strains (figure 3*c* and electronic supplementary material, figure S2).

Previous reports showed that high acidity results in the denaturation or aggregation of periplasmic proteins, and bacteria can induce several factors that counteract the low pH environment [31–33]. We hypothesized that the periplasmic damage in the *ΔpldA* mutant might upregulate factors that would confer resistance to low pH. We therefore measured the acid stress sensitivities of both strains. Cultures of the WT and *ΔpldA* mutant strains were incubated for 20 or 40 min at 37°C at either pH 2.0 or pH 3.0, and cell viability was determined after measuring bacterial colonies on LB plates. While both WT and mutant strains showed minimal survival at pH 2.0 following 40 min incubation, the *ΔpldA* mutant exhibited better survival than the WT strain at pH 2.0 for 20 min. Furthermore, the survival rate of the *ΔpldA* mutant was at least an order of magnitude higher than that of the WT strain after either 20 or 40 min incubation at pH 3.0 (figure 3*d*,*e*), suggesting that the loss of PldA contributes to the higher resistance of *S. flexneri* to acid conditions. In summary, alterations of the antibiotic and acid resistance profile of the *ΔpldA* mutant strain indicate that PldA affects the periplasmic organization of *S. flexneri*. Considering that the *pldA* deletion affects OM integrity (figure 2; electronic supplementary material, table S3), it is likely that this periplasmic disorganization also reflects the dysfunctional bacterial OM.

### 2.4. Transcriptional profiling of wild-type versus *ΔpldA* mutant *Shigella flexneri* strains

**Figure 4.**
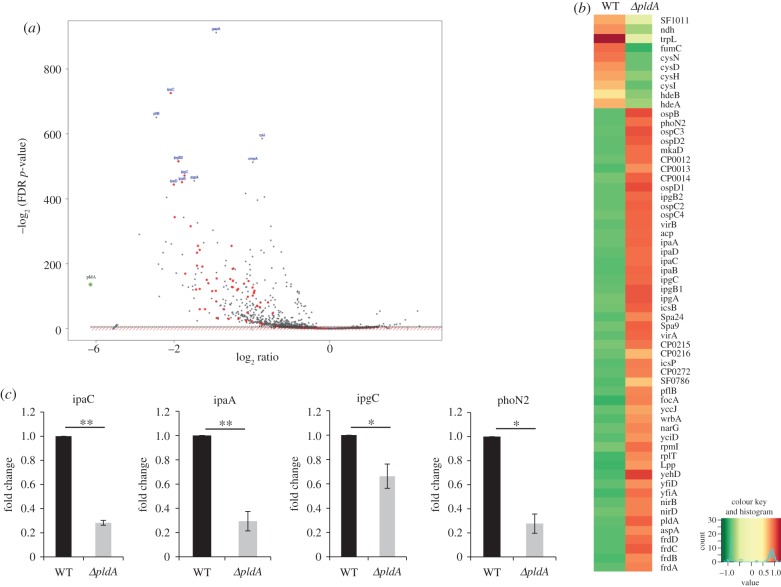
Comparison of *S. flexneri* WT and *pldA* knockout strain transcriptomes. (*a*) Volcano plot showing a differentially expressed genes after *pldA* abrogation (*ΔpldA*/WT). The data are expressed as a log2-fold change in gene expression levels (*x*-axis) plotted against the –log2 *p*-value (*y*-axis). The red dots represent the T3SS-related genes downregulated in the *ΔpldA* mutant. The *pldA* gene is marked by a green circle in the bottom left of the figure. (*b*) Heatmap of RNA sequencing comparing protein expression in the supernatants from WT and *ΔpldA* strains. Gene expression counts were log2 transformed to identify consistent changes in expression profiles between strains. The samples were sequenced in duplicate. (*c*) Quantitative real-time PCR verification of differentially expressed genes in the RNA-seq assay. Total RNA was prepared from the *S. flexneri* WT and *ΔpldA* strains, and expression levels of *ipaC*, *ipaA*, *ipgC* and *phoN2* were analysed. The 16S rRNA expression level was evaluated as an internal control. RNA-seq was performed in duplicate on RNA from exponential phase cultures. qRT-PCR was repeated three times, and the expression level for each gene was normalized to the WT strain. The error bars represent ±s.e.m. (*n* = 3). *p < 0.05; **p < 0.01.

To identify the pathways and genes regulated by *S. flexneri* PldA, we used an RNA-seq-based approach to compare the transcription profile differences between WT and *ΔpldA* strains. The total number of high-quality (Q20) reads generated for each sample ranged from 5.7 to 10.6 million, of which approximately 99% of the reads were successfully mapped to the genome of *S. flexneri*. An overview of the results obtained from the transcriptome is shown in figure 4*a*,*b* and electronic supplementary material, table S4. A comparison of the *ΔpldA* and WT strains revealed that the expression of the 218 *S. flexneri* genes examined in this analysis were significantly downregulated (fold change > 2) after *pldA* deletion. Among these genes, 57 genes were associated with the T3SS apparatus of this pathogen (red dots in figure 4*a*). For example, *ipaA*, *ipaC*, *ipgC* and *ipaD*, which, respectively, serve as T3SS machinery or effectors, were downregulated in the *ΔpldA* strain. This result indicates that PldA is required for the maintenance of a functional T3SS, which mediates the pathogenicity of *S. flexneri*. To validate these results, we further measured the expression levels of selected T3SS genes using quantitative RT-PCR (figure 4*c*). Similar to the results of the transcriptomic assay, we observed that the four targets (*ipaC*, *ipaA*, *ipgC* and *phoN2*) selected for analysis were also downregulated in the *ΔpldA* strain. Taken together, these data indicate that a mutation in *pldA* impacts membrane stability which in turn impacts regulatory networks controlling the expression of T3SS components and associated effectors.

### 2.5. *Shigella flexneri* PldA regulates the T3SS and the bacterial invasion of epithelial cells

**Figure 5.**
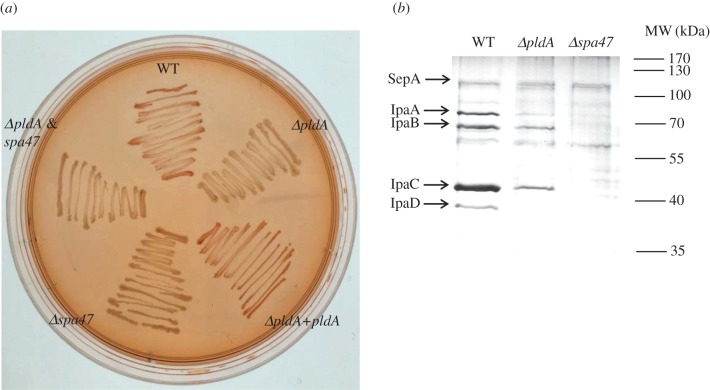
Impact of PldA on the T3SS of *S. flexneri*. (*a*) Various *S. flexneri* strains were grown on a TSA plate containing 0.003% Congo Red. The colony colour was observed after overnight growth. (*b*) The secretome of *S. flexneri* strains were detected by silver staining after Congo Red induction. Equal amounts of total proteins for each sample were loaded for SDS-PAGE. The significant protein bands of interest were identified by MS and are annotated on the left.

Transcriptomic data suggest that PldA may contribute to the functions of *S. flexneri* T3SS. To examine this finding, several bacterial strains were grown on a Congo Red TSA plate (figure 5*a*), showing that the Congo Red-positive phenotype in the WT strain could not be observed in the *pldA*, *spa47* (ATPase for T3SS) or *pldA*&*spa47* mutant strains. Trans-complementation of *pldA* in the *ΔpldA* strain restored the red colour on the Congo Red plate. Further experiments were conducted to validate the influence of PldA on T3SS secretion. The secretome of WT, *ΔpldA* and *Δspa47* strains in the presence of Congo Red was subsequently monitored. As shown in figure 5*b*, although the secretion of SepA, which is an autotransporter independent of the T3SS, was not affected, the secretion of major effectors of T3SS, including IpaA, IpaB, IpaC and IpaD, were all significantly downregulated after *pldA* deletion. Also, much of the T3SS-related proteins were undetectable in the *ΔpldA* mutant by MS analysis (electronic supplementary material, table S5). As for the negative control, these T3SS effectors could not be detected in the *Δspa47* or *ΔpldA&spa47* supernatant (figure 5*b* and electronic supplementary material, table S5). Hence, these results showed that PldA significantly impacts the T3SS function in *Shigella*.

**Figure 6.**
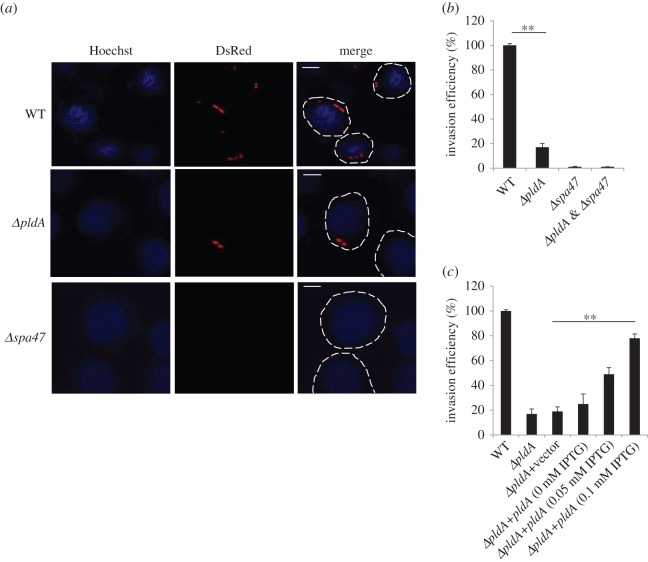
Deletion of *pldA* affects the internalization of *S. flexneri* into HeLa cells. (*a*) Confocal microscopy analysis of HeLa cells infected with *S. flexneri* WT, *ΔpldA* or *Δspa47* strains. *Δspa47* (the T3SS-deficient strain) served as a negative control. Scale bars, 5 µm. (*b*) The invasion efficiency of the *S. flexneri* WT, *ΔpldA* or *Δspa47* strains into HeLa cells was assayed after infection. (*c*) The bacterial invasion assay of *ΔpldA* strain complemented with *pldA* gene. Different concentrations of IPTG (0 –0.1 mM) were supplemented to determine the optimal invasion conditions. The invasion percentage of indicated strains is normalized to the WT, and the error bars represent ±s.e.m. (*n* = 3), **p < 0.01.

Because T3SS plays a critical role in the pathogenesis of *Shigella*, the effect of PldA upon the internalization of *S. flexneri* was also investigated. We infected human epithelial HeLa cells with various DsRed-expressing bacterial strains. As shown in figure 6*a*, we observed fewer bacteria in host cells infected with the *ΔpldA* strain compared with those infected with the WT strain. We used the *Δspa47* strain, which lacks a functional T3SS, as a negative control; this mutant was unable to infect HeLa cells (figure 6*a*). To validate the relationship between PldA and the pathogenicity of *S. flexneri* using another approach, we performed gentamicin protection assay during host cell infection. We infected HeLa cells with *S. flexneri* strains in the exponential phase and measured the internalized colony-forming units (CFUs). We observed that the invasion ability of the *ΔpldA* mutant dramatically decreased compared with the WT strain (figure 6*b*). As a negative control, the *Δspa47* and *ΔpldA&spa47* strains displayed no invasion efficiency (figure 6*b*). To establish that PldA directly affected invasion, we complemented the *ΔpldA* mutant PldA via an IPTG (isopropyl-beta-d-thiogalactopyranoside)-inducible plasmid. The invasion efficiency increased in an IPTG concentration-dependent manner up to 0.1 mM IPTG (figure 6*c*). Additionally, we observed no significant difference in cell adhesion and cytotoxicity as measured based on the LDH release among the strains examine above (electronic supplementary material, figure S3*a*,*b*). It is likely that *S. flexneri* PldA regulates host cell invasion and cellular internalization through the manipulation of T3SS functions.

### 2.6. Intracellular *Shigella flexneri* delivers PldA into the epithelial cell cytosol

**Figure 7.**
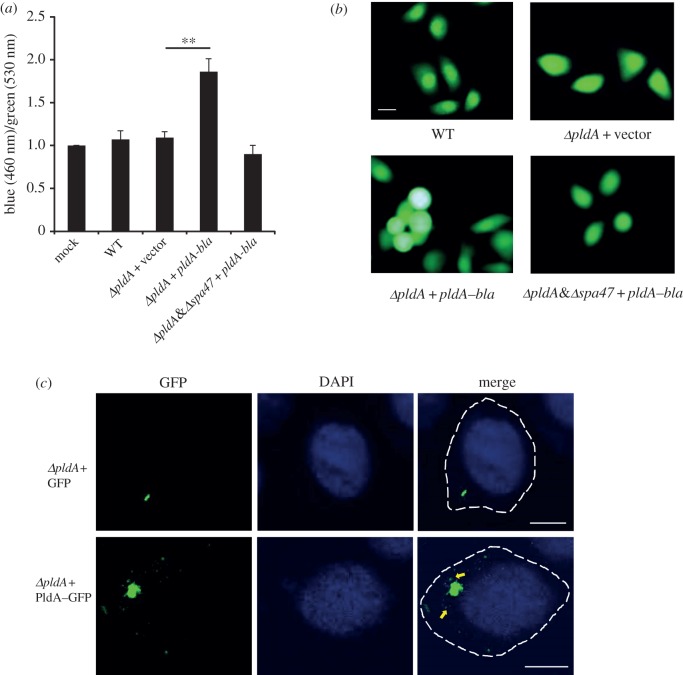
Secretion of PldA–Bla inside HeLa cells. (*a*) Analysis of the secretion of PldA–Bla fusions from *Shigella* strains into HeLa cells. HeLa cells were infected with the indicated strains expressing TEM-1 fused with PldA and then treated with CCF2-AM after infection. β-Lactamase activity in HeLa cells is expressed as the ratio of blue (460 nm) and green fluorescence (530 nm) corresponding to the cleaved and uncleaved CCF2 products, respectively, normalized to mock. The error bars represent ±s.e.m. (*n* = 3), **p < 0.01. (*b*) Visualization of the secretion of PldA by fluorescence microscopy. HeLa cells infected with the indicated *Shigella* strains were loaded with CCF2-AM. Green cells contain intact CCF2-AM, whereas blue cells contain cleaved CCF2-AM, corresponding to the translocated PldA–Bla fusion proteins. Scale bar, 10 µm. (*c*) *ΔpldA* strain producing GFP or PldA–GFP was applied to infect HeLa cells. The cells were subsequently fixed and examined by confocal microscopy. The yellow arrows in the bottom image indicate the green particles containing PldA–GFP, secreted away from the bacterial cells. No such particles can be observed in the strains producing the GFP-only control. The images are representatives of three independent experiments.

In addition to the functions of PldA in the prokaryotic cell, the performance of this enzyme inside the eukaryotic host was also investigated. A previous study showed that the intracellular PldA levels significantly increased during the infection of HeLa cells with *S. flexneri* [29]. We therefore assumed that PldA might also play a direct role in the eukaryotic cell invasion and delivery of PldA into HeLa cells, assayed using β-lactamase (Bla) fused with PldA. Epithelial cells infected with the *S. flexneri* strain expressing the PldA–Bla fusion protein were treated with CCF2-AM, a fluorescent β-lactamase substrate that emits blue fluorescence after cleavage with β-lactamase. Consistently, HeLa cells infected with the *S. flexneri ΔpldA* strain expressing the PldA–Bla fusion protein exhibited higher blue/green fluorescence ratios than those infected with WT strain or with the *ΔpldA* mutant strain (figure 7*a*,*b*). Additionally, the fluorescence ratio of *ΔpldA&spa47* double mutant expressing *pldA–bla* decreased to the basal level of mock control, suggesting that T3SS is important in the process of PldA transport. The PldA secretion into the host cell was further validated by confocal microscopy (figure 7*c*). After infection with *S. flexneri* harbouring plasmids expressing *pldA–gfp*, HeLa cells were fixed and examined using confocal microscopy. The GFP signal was observed in the cytosol around the internalized bacteria. As a negative control, cells infected with *S. flexneri* producing GFP protein alone did not exhibit any scattered green signal. Thus, these results indicate that intracellular *S. flexneri* secretes PldA into the host cell cytosol.

### 2.7. PldA homologues are widely distributed in bacteria

**Figure 8.**
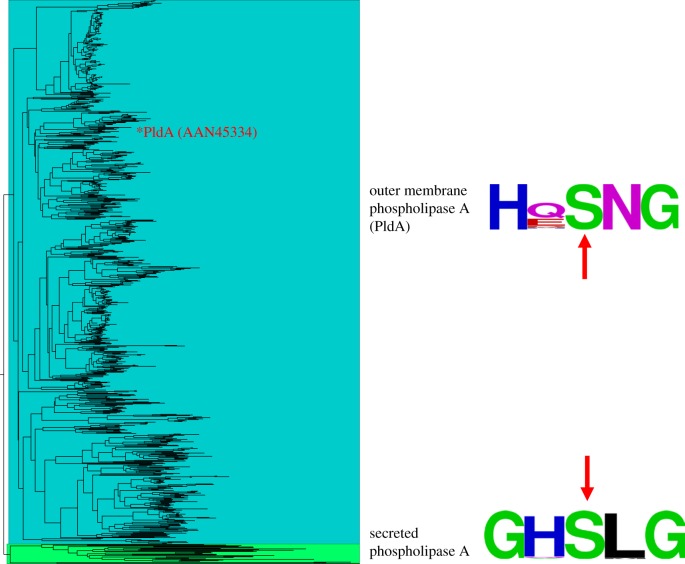
Maximum-likelihood phylogenetic tree of 858 bacterial phospholipase A proteins. Clades of OMPLA (PldA) proteins and secreted phospholipase A proteins are highlighted in blue and green, respectively. Sequence logos of the conserved catalytic motifs for both groups are provided accordingly. The location of PldA (asterisk) and the active site of the catalytic domain (arrow) are indicated.

The phospholipase A protein family in bacteria comprises a large number of hydrolases, which play a critical role in virulence [17]. A variety of phospholipase A effectors, such as *yplA* in *Yersinia enterocolitica* and *phlA* in *Serratia marcescens*, were secreted from the pathogens [34–38]. We conducted a phylogenetic analysis to examine the membrane bound and secreted phospholipase A. Using an exhaustive search in the public domain, we identified PldA homologues among 828 bacteria species of 284 genera, among which the majority (84%) are bacteria from the Proteobacteria phylum, the largest group of Gram-negative bacteria. And 26 genera of these organisms also possess T3SS (electronic supplementary material, table S6). Phylogenetic analysis groups all PldA homologues in a distinct clade from secreted phospholipase A proteins (figure 8). Indeed, although the overall sequence similarities between different PldA homologues were as low as approximately 13%, the catalytic motif of phospholipase A was highly conserved with an *HxSNG* pattern in all bacteria (figure 8). By contrast, the secreted phospholipase A proteins, located in a distinct clade, shared a *GxSxG* catalytic motif as suggested in a previous study [39].

## 3. Discussion

PldA is widely distributed and well conserved in Gram-negative bacteria, indicating that this enzyme may play an important role in bacterial survival. In *E. coli*, PldA is a constitutively expressed, predominantly outer membrane protein with phospholipase A activity and is considered as a house-keeping gene [17]. While there have been several reports addressing the PldA function under stress conditions, there is less attention on the effects of this enzyme during homoeostasis. In this study, we report a comprehensive validation of how *S. flexneri* PldA functions under standard growth conditions using proteomic and transcriptomic approaches. Furthermore, we define the role of this enzyme as a virulence determinant during *S. flexneri* internalization into epithelial host cells.

A biophysical model showed that PldA affected the asymmetric OM under normal conditions [13,14]. Here, we characterized the extracellular proteome for both *S. flexneri* WT and *ΔpldA* strains during the exponential growth phase through MS to detect the membrane integrity and cellular content leakage. The results showed that 149 proteins were exclusively present in the extracellular proteome of the *ΔpldA* strain and suggest that the PldA disruption either directly or indirectly leads to the damage of bacterial inner and/or outer membrane and the subsequent secretion of additional proteins into the culture supernatant. PldA deficiency affected proteins from multiple functional categories, such as the ABC (ATP-binding cassette) transporter and associated substrate binding proteins (ArtI, OppA, PotD, HisJ, PstS and YrbC) located in the periplasm, which undergo conformational changes upon substrate binding in the bacteria. Therefore, PldA may regulate the physiological function of ABC transport systems. The culture supernatant of *ΔpldA* strain also showed elevated levels of BamC (NlpB) and BamD. In Gram-negative bacteria, the BAM (β-barrel assembly machinery) complex is responsible for the folding and insertion of β-barrel proteins into the OM [40]. In *Salmonella*, BAM is also involved in the OMP biogenesis and the expression of the T3SS [41]. Together, the loss of PldA would disrupt the bacterial OM through multiple mechanisms and confer adverse effects on the virulence of the pathogen.

The important roles of PldA in stabilizing cell wall integrity could explain the phenotypes we observed. In the absence of OM protection, proteins in the periplasmic space would become denatured or aggregate without causing cell death (electronic supplementary material, figure S1*b*). Penicillin-binding proteins (PBPs) are a group of proteins essential for bacterial peptidoglycan biogenesis in the periplasm and are binding targets of β-lactam antibiotics [42]. The dysregulation in PBP conformation would lead to the reduced affinity of PBPs for β-lactam antibiotics and consequently result in resistance to these types of antibiotics in bacteria. The best-characterized bacterial form containing deficient cell wall architecture is termed the L-form and can be observed in both Gram-positive and Gram-negative species. These bacteria are indeed osmotically sensitive and generally resistant to β-lactam antibiotics, reflecting cell wall perturbations. Additionally, in *Listeria monocytogenes*, stable L-forms exhibit the dramatic attenuation of pathogenicity towards host cells [43]. These L-form properties are similar to those we observed in the *S. flexneri ΔpldA* mutant.

The observation that the *ΔpldA* strain displayed increased viability upon acid stress might also reflect OM defects. Two periplasmic proteins (HdeA and HdeB) were defined as key factors in bacterial acid resistance [31–33]. These proteins serve as chaperones and prevent the aggregation of periplasmic proteins exclusively below pH 3. We reasoned that the denaturation status of periplasm proteins in the *ΔpldA* strain caused by OM changes led to the upregulation of *hdeA* and *hdeB* genes and thereby enhanced the acid resistance capacity of the *ΔpldA* strain. The results of RNA-seq validated this hypothesis, as the transcription levels of *hdeA* and *hdeB* were significantly upregulated in the *ΔpldA* strain (figure 4*b*).

To identify genes regulated by PldA, we investigated the transcriptome profiles of *S. flexneri* WT and *ΔpldA* strains using high-throughput RNA-seq technology. RNA-seq results demonstrated that PldA greatly influences the transcription levels of genes encoded in both the bacterial genome and in the virulence plasmid. A number of virulence-associated loci were downregulated in the *ΔpldA* mutant, including *ipaC*, *ipgC*, *ipaB*, *ipaA* and the virulence gene transcriptional activator *virB*. Given that the secretion of these effectors was also downregulated in the *ΔpldA* mutant, we speculate that the disorganized OM structure and the altered periplasmic space of the *ΔpldA* mutant might hinder the precise assembly and secretion of the T3SS apparatus, and consequently the decreased invasion ability. In addition, significant transcriptional changes were observed for genes involved in various metabolic pathways. According to the KEGG pathway analysis (http://www.kegg.jp/), the most downregulated genes in the *ΔpldA* mutant strain were associated with nitrogen metabolism (*nirB*, *nirC*, *nirD*, *narG*, *narH* and *narJ*), carbon metabolism (*frdA*, *frdB*, *frdC* and *frdD*) and osmotic response (*osmC* and *osmE*). PldA deficiency also resulted in the downregulation of OMP gene transcripts (*ompA* and *ompX*). By contrast, genes encoding acid resistance proteins (*hdeB* and *hdeA*) and sulfur metabolism-related proteins (*cysN*, *cysD*, *cysI* and *cysH*) were upregulated by PldA deficiency. For pathogens to efficiently survive and replicate in the host cell, it is important that these organisms coordinate their metabolic system to adapt to the host cell physical conditions [44,45]. Furthermore, several OMPs regulated by PldA were associated with pathogen virulence. In *S. flexneri*, OmpA plays a role in protrusion formation and inter-cellular spreading [5]. In conclusion, the expression of several genes encoding T3SS-related proteins, metabolism-related proteins and OMPs are disrupted in the *pldA* knockout mutant, consistent with the finding that the *ΔpldA* strain exhibited decreased invasion capacity into HeLa cells.

Gram-negative bacteria possess several secretion mechanisms for translocating virulence factors. A particularly important mechanism is vesiculation, where the outer membrane vesicles (OMVs) bud from the OM; these vesicles contain the OM and entrapped periplasmic contents [46]. Various OMPs were observed in the OMV proteomes from bacteria, such as *Pasteurella multocida* [47] and *Vibrio cholerae* [48]. Through proteomic analysis, PldA was detected in the OMV of *S. flexneri* [49]. Using a TEM-1 fusion PldA and confocal microscopy observation, we determined that PldA is delivered into HeLa cell cytosol in the presence of functional T3SS after bacterial internalization. As PldA is not a T3SS effector but is abundantly expressed intracellularly [29], it is possible that PldA may be induced for proper membrane organization in intracellular conditions and/or delivered through additional mechanisms during the intracellular growth of *Shigella*. This latter phenomenon might be accomplished via OMV production. In *Legionella pneumophila*, the secreted effector VipD with phospholipase A1 activity binds Rab5 and Rab22 in host cell endosomes and consequently protects the bacteria from endosomal fusion [50,51]. Therefore, following infection, the PldA from *S. flexneri* may also participate in the interaction between the bacteria and the host cell. Future work is needed to elucidate how PldA interacts with the host factors within the cell cytoplasm.

The phospholipase A proteins are widely distributed in species ranging from bacteria to mammals [39]. Hence, the potential roles of PldA protein in both prokaryotic and eukaryotic cells are intriguing. In prokaryotic cells, PldA is required for stabilizing the bacterial cell wall structure. The loss of this protein in the OM of *S. flexneri* results in dysregulated bacterial morphology, cytoplasmic content leakage, alteration of periplasmic space and, eventually, the attenuation of pathogenicity. However, PldA might also interact with eukaryotic host cells in a similar manner as its counterparts in *Serratia* spp*.* or *Y. enterocolitica* [39]. Indeed, PldA is present in the OMVs of *S. flexneri* [49], and the expression level of this protein is greatly elevated after *S. flexneri* internalizes into epithelial cells [29]. These findings suggest that PldA may also be functional in mediating bacterial pathogenicity.

## 4. Material and methods

### 4.1. Bacterial strains, plasmids and growth conditions

Bacterial strains and plasmids used in this study and their relevant characteristics are listed in the electronic supplementary material, table S1. All strains were routinely cultured at 37°C in Tryptic Soy Broth (TSB) and Luria-Bertani (LB) medium (for *S. flexneri* and *E. coli*, respectively). LBG medium (buffered LB medium containing 0.4% glucose) was used in the acid resistance assay. Antibiotic supplements were used at the following concentrations: 25 µg ml^−1^ chloramphenicol for *S. flexneri* (except for the antibiotic sensitivity assay, in which the appropriate antibiotics were diluted to the indicated concentrations), and 25 µg ml^−1^ chloramphenicol, 100 µg ml^−1^ ampicillin and 25 µg ml^−1^ kanamycin for *E. coli*. Plasmid pKO3 was used to construct *S. flexneri pldA* and *pldA&spa47* deletion mutants as described previously [52]. In-frame chromosomal deletion of *pldA* and *spa47* genes was constructed according to the pKO3 gene replacement protocol.

### 4.2. DNA manipulation and plasmid construction

Standard protocols were used for PCR amplification and for bacterial transformation as described [53]. DNA amplification was carried out using Q5 high-fidelity DNA polymerase (New England Biolabs). *Escherichia coli* strain DH5α was used for cloning; *pldA* gene was amplified from *S. flexneri* 2a str. 301 genome, cloned into pVTRA' expression vector with a 3′ hexahistidine tag for complementation expression. For the construction of *pldA–gfp* or *pldA–bla*, amplicons of *pldA*, *gfp* and *bla* were generated and overlap PCR was applied to produce the recombinant *pldA–gfp* or *pldA–bla* fragment (*pldA* in 5′ ends). The products were then ligated into the pVTRA' vector for expression.

### 4.3. SDS-EDTA permeabilization assay

Experiments were carried out as described previously [54]. Briefly, bacterial cells from overnight culture were sub-inoculated into TSB broth and grown to exponential phase. Cells were subjected to treatments with 1.5 mM EDTA for 20 min at 37°C in the microtitre plate wells. The wells were pre-loaded with 0.5% SDS or buffer only controls. Turbidity of the cell suspensions was monitored with a microplate reader.

### 4.4. Antibiotic sensitivity assay

After overnight (20 h) growth in the TSB medium, cultures of *S. flexneri* WT and *ΔpldA* strains were sub-cultured and grown with aeration at 200 r.p.m. When grown to exponential phase (OD600 ∼ 0.5), cultures were diluted 1 : 200 and added into the TSB media with titrated antibiotics in 96-well plates. The cultures were then grown at 37°C without shaking. OD600 was recorded after 24 h growth.

### 4.5. Acid resistance assay

After overnight (20 h) growth in the LBG medium (pH 7.0), cultures of *S. flexneri* WT and *ΔpldA* strains were sub-cultured and grown with aeration at 200 r.p.m. When grown to exponential phase an aliquot of the culture was diluted 1 : 100 into the LBG medium at the indicated pH and incubated at 37°C for 20 or 40 min. The cells were diluted in the LB medium, plated on LB agar and incubated at 37°C for 15 h before colony counting. As a control, the overnight culture grown at regular pH was diluted in LB medium, plated on LB agar and incubated at 37°C for 15 h. Colonies were counted to calculate the percentage survival of *S. flexneri*.

### 4.6. Cell invasion assays

HeLa cells were cultured in Dulbecco's modified Eagle's medium (DMEM, Thermo Scientific) supplemented with 10% fetal bovine serum (FBS) on 12-well plates. When grown to 70% confluence at 37°C (5% CO_2_), cells were washed with sterile PBS and infected with the indicated *S. flexneri* strains from exponential phase culture at a multiplicity of infection (MOI) of 50 : 1. Following infection for 30 min, HeLa cells were washed and treated with 50 µg ml^−1^ gentamicin for 2 h. Finally, cells were permeabilized with 0.1% Triton X-100 on ice for 30 min. After serial dilution, CFUs were counted to determine the number of internalized bacteria in the epithelial cells.

### 4.7. Confocal microscopy

HeLa cells were cultured overnight in DMEM with 10% FBS. Following bacterial infection for 30 min, cells were washed, treated with gentamicin, fixed with 4% paraformaldehyde, stained with DAPI or Hoechst (Sigma) and examined by microscopy. Images were captured using a Leica TCS SP5 confocal microscopy.

### 4.8. Outer membrane purification

Overnight cultures of *S. flexneri* WT and *ΔpldA* strains were sub-cultured and grown to exponential phase with aeration at 200 r.p.m. in 10 ml TSB medium. Bacteria were then harvested (4000 r.p.m. centrifugation for 10 min), and the OM was purified according to the protocol previous described [55].

### 4.9. Mass spectrometric analysis

Overnight cultures of *S. flexneri* WT and *ΔpldA* strains were sub-cultured into the TSB medium, and grown to exponential phase. Cultures were pelleted by centrifugation at 4000*g* for 10 min. To prepare the Congo Red induced supernatant, 0.003% Congo Red was added when grown to exponential phase and culture was harvested after 1 h induction. The culture supernatants were passed through 0.2 µm filters to eliminate the residual *S. flexneri*, and the extracellular proteins were collected by precipitation with 10% trichloroacetic acid. In-gel protein digestion and MS analysis were completed according to the protocol as described previously [56]. An LTQ Orbitrap Velos mass spectrometer (Thermo Fisher Scientific, Germany) was used for MS/MS spectra analysis. The raw data from the analysis were processed using Proteome Discoverer software (v. 1.4.1.12; Thermo Fisher Scientific, Waltham, MA, USA) with two different search algorithms, MASCOT (v. 2.3.02, Matrix Sciences, UK) and SEQUEST (Thermo Fisher Scientific). The MS/MS spectra were searched against the *Shigella* protein database from NCBI (RefSeq NC_004337.2 and NC_004851.1). All of the raw mass spectra files and merged peak list files in this study have been deposited into the publicly accessible database PeptideAtlas and are available under dataset Identifier PASS00838. The subcellular locations of the identified proteins were predicted using the bioinformatic algorithms PSORTb, SignalP and TMHMM and their functional annotations categorized according to the KEGG pathway analysis (http://www.kegg.jp/).

### 4.10. RNA sequencing (RNA-seq) and data analysis

Total RNA was extracted from exponential cultures of *S. flexneri* WT and *ΔpldA* strains. RNA was isolated with the RNeasy Mini kit (Qiagen) and eluted with 20 µl of RNAse-free water. DNA was removed by TURBO DNA-free Kit (Ambion), and rRNA depleted by Bacteria Ribo-Zero rRNA removal kit (Epicentre) according to the manufacturer's instructions. Double strand cDNA was synthesized using the PrimeScript Double-Strand cDNA Synthesis Kit (TaKaRa). DNA quantity and quality was assessed by Agilent 2100 BioAnalyzer. High-throughput sequencing was performed using an Illumina HiSeq 2500 sequencer (single end, 101 bp read length). Samples were prepared and sequenced in duplicate. All of the raw data have been deposited in the Sequence Read Archive (SRP071559). Sequencing data are summarized in the electronic supplementary material, table S4.

### 4.11. Quantitative RT-PCR analysis

Total RNA was extracted and DNA removed by TURBO DNA-free Kit (Ambion), and cDNA was reverse-transcribed using SuperScript VILO Master Mix (Invitrogen). Quantitative RT-PCR was performed using Power SYBR Green PCR Master Mix (Applied Biosystems) and the values normalized to 16S rRNA. RNA expression was quantitatively measured using the Livak (2^−ΔΔCT^) method.

### 4.12. Growth curve analysis

Overnight cultures of *S. flexneri* WT and *ΔpldA* strains were sub-cultured into the TSB medium and grown with aeration at 200 r.p.m. OD600 was recorded at different time points to evaluate growth rate. At each time point, the bacterial culture was serially diluted in TSB and spread onto a TSA plate for viable colony number counting.

### 4.13. Cell adhesion and cytotoxicity assays

HeLa cells were used in both assays. Adhesion assays were performed using a procedure similar to the cell invasion assay; however, gentamicin incubation was omitted. For cytotoxicity assays, cells were cultured on 96-well plates in DMEM medium deprived of sodium pyruvate. After 1 h infection with the indicated bacteria at an MOI of 50 : 1, the cytotoxicity was measured using CytoTox-ONE homogeneous membrane integrity assay kit (Promega).

### 4.14. Translocation assay

HeLa cells were grown on 12-well plates to 70% confluence, washed twice with PBS, then infected with the bacterial strains expressing PldA–Bla fusion proteins (at an MOI of 50 : 1) for 30 min. After infection, cells were washed twice with HBSS and treated with CCF2-AM (Invitrogen) for 90 min at room temperature. Fluorescence was quantified using a microtitre plate reader following excitation at 405 nm according to the manufacturer's instructions. Translocation was expressed as a ratio of signals obtained from cleaved (460 nm, blue) and uncleaved (530 nm, green) fusion proteins. Translocation was further assessed using a Nikon fluorescence microscope.

### 4.15. Bioinformatic analysis

Bacterial homologues of PldA were extracted from the non-redundant protein database (NR) using the position-specific iterated BLAST algorithm (PSI-BLAST) available from the NCBI website [57]. The PldA sequence of *S. flexneri* 2a str. 301 (GenBank accession: AAN45334.1) was used as the initial query for PSI-BLAST. The iterated searches were limited to Bacteria domain with a maximum number of hits to 10 000 per round. A total of 3599 homologues were retrieved by PSI-BLAST with a statistical significance threshold of 0.005 before the iterations reported no additional valid hits. Then all PldA homologues were downloaded and screened to exclude sequences without explicit taxonomic information (i.e. those from uncultured or environmental samples). For brevity, only one PldA homologue of each bacterial species (the best hit) was kept for further analysis. Two well-studied secreted phospholipase A proteins from *Yersinia enterocolitica* and *Serratia proteamaculans* were initially collected based on the original literature [34,35,37]. Representative homologues of the two secreted phospholipase A proteins from other bacteria species were manually selected based on the BLASTP searches amongst the NR databases. The final bacterial phospholipase A dataset includes 828 PldA homologues and 30 secreted phospholipase A proteins. Multiple sequence alignment was conducted with MUSCLE 3.8 [58]. Maximum-likelihood phylogenetic tree was then inferred from the alignment using FastTree 2.1 with default parameters [59]. Sequence logos for the catalytic motifs of phospholipase A were produced by WebLogo 2 [60].

### 4.16. Statistical analysis

A two-tailed Student's *t*-test was used to confirm statistical significance at 95% confidence between the two samples compared. A *p*-value of less than 0.05 was considered to be significant.

## Supplementary Material

Supplemental figures 1-3

## Supplementary Material

Supplemental table 1

## Supplementary Material

Supplemental table 2

## Supplementary Material

Supplemental table 3

## Supplementary Material

Supplemental table 4

## Supplementary Material

Supplemental table 5

## Supplementary Material

Supplemental table 6

## Data accessibility

The RNA-seq raw data have been deposited in the Sequence Read Archive (SRP071559). The raw mass spectra files and merged peak list files have been deposited into the publicly accessible database PeptideAtlas (PASS00838).
