# Supplementary material for: The outer membrane phospholipase A is essential for membrane integrity and type III secretion in Shigella flexneri
Source: Open Biol. 2016 Sep 21;6(9):160073. doi: 10.1098/rsob.160073 (PMC5043575; doi:10.1098/rsob.160073)
Supplement: Supplemental figures 1-3 [file rsob160073supp1.pdf]

The Outer Membrane Phospholipase A is essential for membrane integrity and Type III Secretion in Shigella flexneri

Xia Wang†, Feng Jiang†, Jianhua Zheng†, Lihong Chen†, Jie Dong, Lilian Sun, Yafang Zhu, Bo Liu, Jian Yang, Guowei Yang, Qi Jin

Figure S1

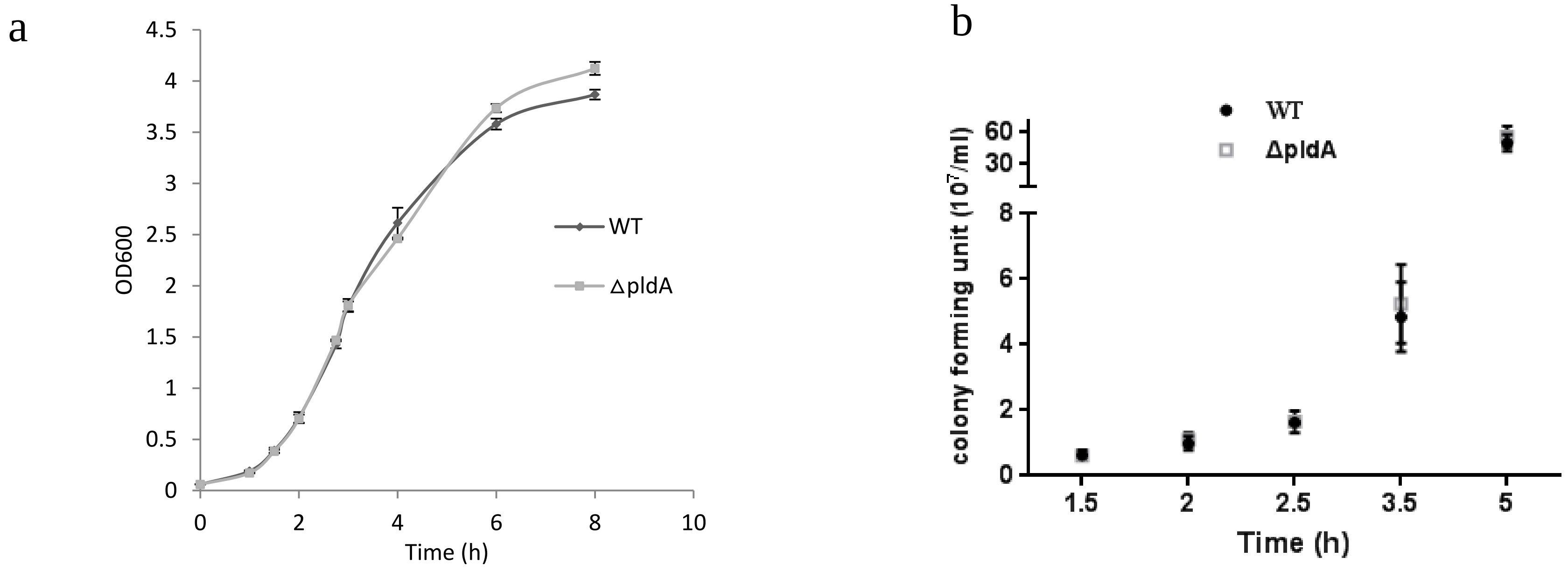

Figure S1 *pldA* deletion did not influence the growth rate of *S. flexneri*. (a) Growth curves of indicated strains in TSB medium at 37°C were shown. (b) Viable colony numbers of WT and *ΔpldA* mutant strains at each time point. Data are representative of at least three biological replicates.

Figure S2

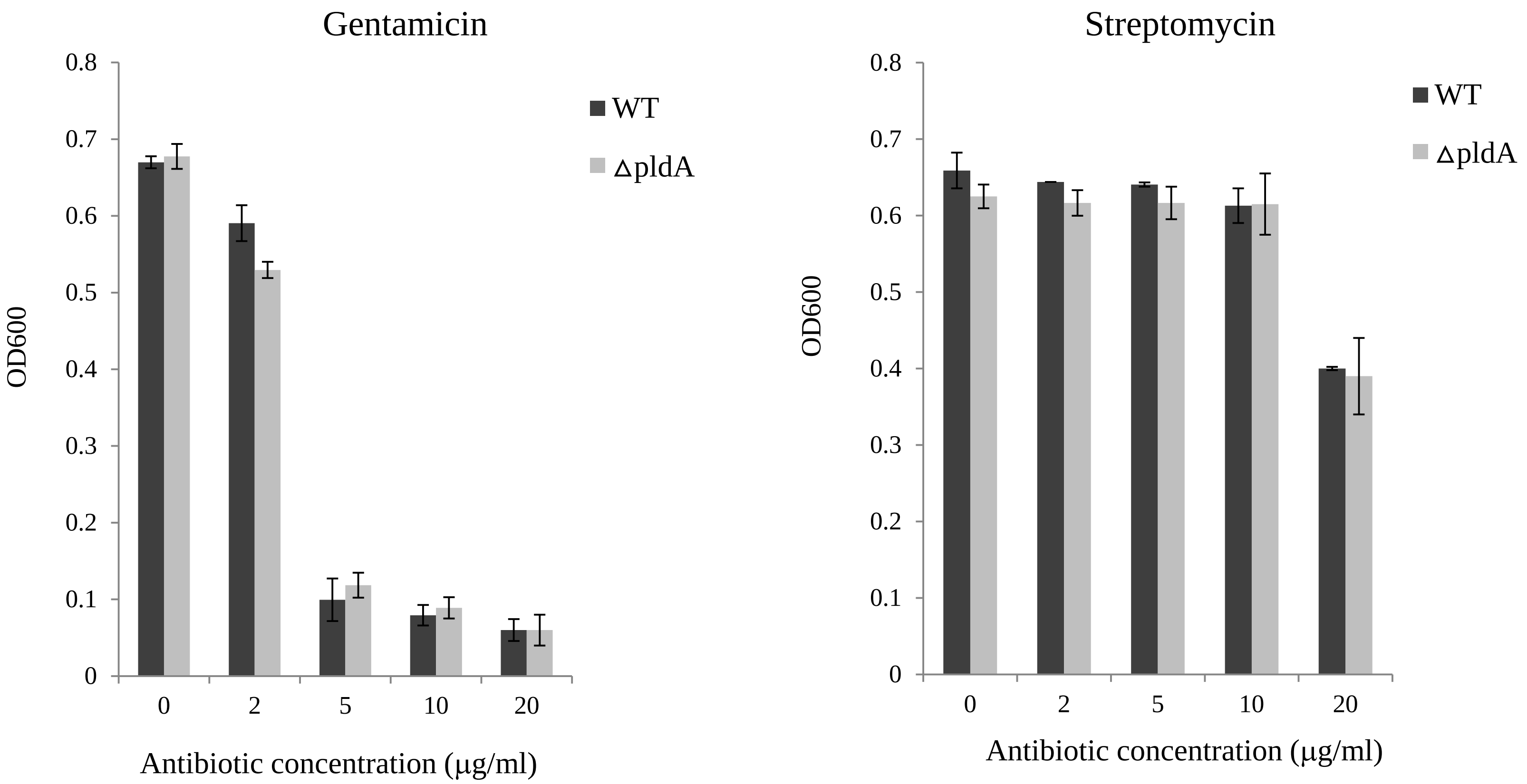

Figure S2 non-β-lactam antibiotic resistance profile in *S. flexneri* WT and *ΔpldA* strains. The OD600 of indicated *S. flexneri* strains after 24 hr growth in the TSB medium in the presence of non-β-lactam antibiotics (gentamicin, streptomycin).

Figure S3

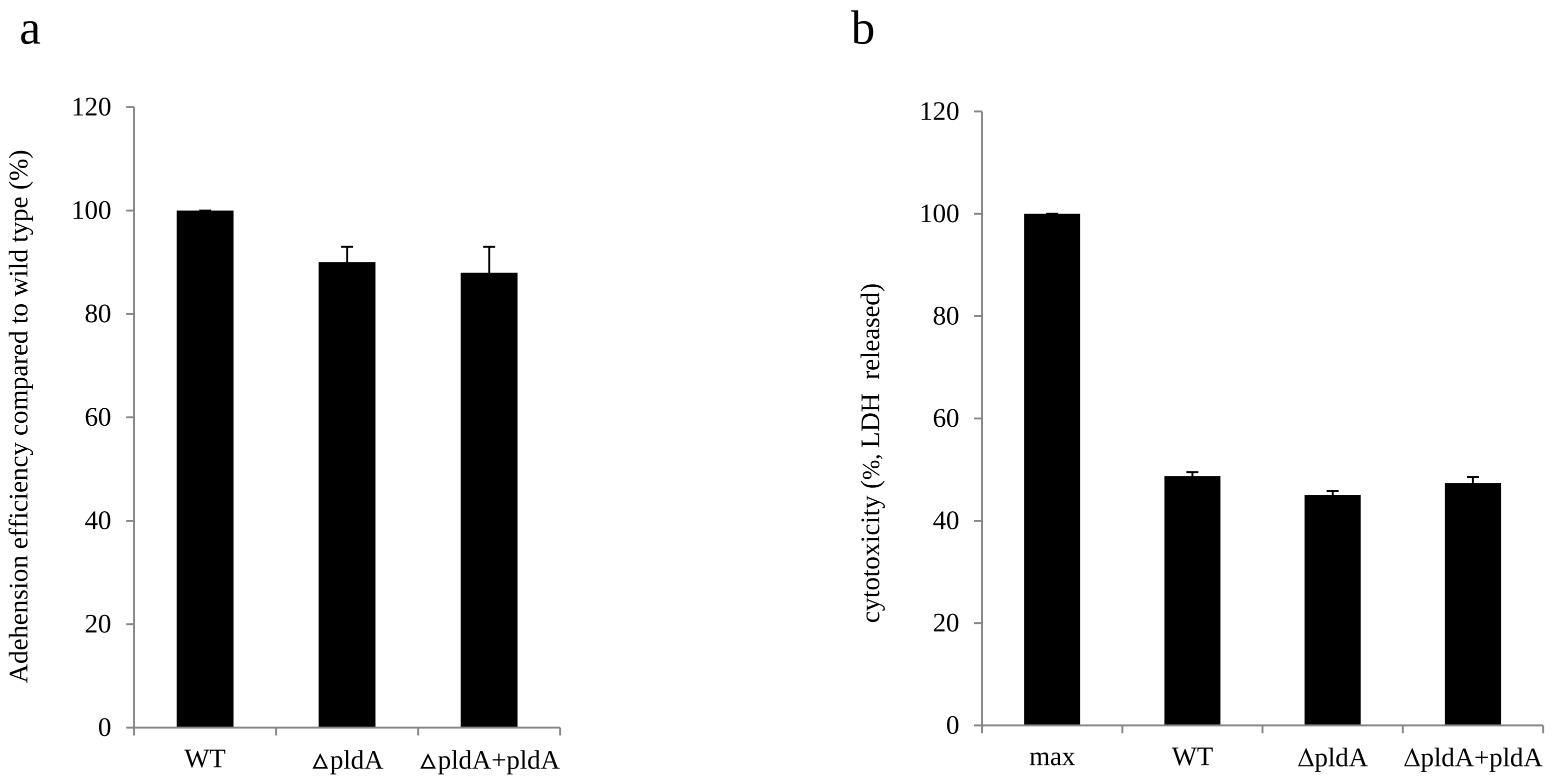

Figure S3 Bacterial adhesion (a) and cytotoxicity (b) assays in HeLa cells. (a) The adhesion efficiency of the indicated S. *flexneri* strains, normalized to the WT strain, confirming that PldA is not involved in the adhension ability to Hela cells. (b) PldA deficiency did not exhibit cytotoxicity to HeLa cells as confirmed by LDH released assays. The maximum LDH release, as a measure of cytotoxicity, was shown using the positive control provided by the manufacturer (Promega). Error bars represent ± SEM (n = 3), and 0.1 mM IPTG was used for the induction of PldA expression in both assays.
