# Supplementary material for: The outer membrane phospholipase A is essential for membrane integrity and type III secretion in Shigella flexneri
Source: Open Biol. 2016 Sep 21;6(9):160073. doi: 10.1098/rsob.160073 (PMC5043575; doi:10.1098/rsob.160073)
Supplement: Supplemental table 1 [file rsob160073supp2.docx]

| **Strains and plasmids used in this study.** | |  |
| --- | --- | --- |
| Strains & plasmids | Description | Source or reference |
| Strains |  |  |
| sf301 | Wild-type *S. flexneri* strain | Lab Stock |
| sf301ΔPCP | *S. flexneri* virulent plasmid deficient strain | Lab Stock |
| sf301Δ*pldA* | Wild-type *S. flexneri* strain depleted of *pldA* gene | This work |
| DH5α | *E.coli* K-12 | Invitrogen |
| Plasmids |  |  |
| pKO3 | repA(Ts) Cm^r^ sacB+ | ([1](#_ENREF_1)) |
| pVTRA' | pSC101 derivative, lacI q-Ptac expression vector, Cm^r^ | ([2](#_ENREF_2)) |
| pVTRA'-pldA | pVTRA' expressing *pldA* gene | This work |
| pVTRA'-pldA-bla | pVTRA' expressing PldA::Bla fusion proteins | This work |
| pBBR-DsRed | Broad host plasmid pBBR1MCS5 expressing fluorescent DsRed protein | Lab Stock |

**The Outer Membrane Phospholipase A is essential for membrane integrity and Type III Secretion in Shigella flexneri**

**Xia Wang†, Feng Jiang†, Jianhua Zheng†, Lihong Chen†, Jie Dong, Lilian Sun, Yafang Zhu, Bo Liu, Jian Yang, Guowei Yang, Qi Jin**

1. Link, A. J., Phillips, D., and Church, G. M. (1997) Methods for generating precise deletions and insertions in the genome of wild-type Escherichia coli: application to open reading frame characterization. *Journal of bacteriology* **179**, 6228-6237

2. Perez-Martin, J., and de Lorenzo, V. (1996) VTR expression cassettes for engineering conditional phenotypes in Pseudomonas: activity of the Pu promoter of the TOL plasmid under limiting concentrations of the XylR activator protein. *Gene* **172**, 81-86
