# Supplementary material for: The outer membrane phospholipase A is essential for membrane integrity and type III secretion in Shigella flexneri
Source: Open Biol. 2016 Sep 21;6(9):160073. doi: 10.1098/rsob.160073 (PMC5043575; doi:10.1098/rsob.160073)
Supplement: Supplemental table 6 [file rsob160073supp7.pdf]

Table S6

Table S6 The genus with co-occurrence of PldA and T3SS in the phylogenetic tree

Genus	PldA	T3SS
Aeromonas, Bordetella, Bradyrhizobium, Burkholderia	+	+
Chromobacterium, Citrobacter, Chlamydia, Chlamydophila	+	+
Desulfovibrio, Escherichia, Edwardsiella, Erwinia	+	+
Mesorhizobium, Pantoea, Parachlamydia, Photorhabdus, Pseudomonas	+	+
Ralstonia, Rhizobium, Salmonella, Shigella, Sinorhizobium, Sodalis	+	+
Vibrio, Xanthomonas, Yersinia	+	+
